# Loss of Heterozygosity for *Kras^G12D^* Promotes Malignant Phenotype of Pancreatic Ductal Adenocarcinoma by Activating HIF-2α-c-Myc-Regulated Glutamine Metabolism

**DOI:** 10.3390/ijms23126697

**Published:** 2022-06-15

**Authors:** Yu Ma, Sunkai Ling, Yuan Li, Mingyue Hu, Bo Kong, Peilin Huang, Hui Liu

**Affiliations:** 1 Department of Pathology, Xuzhou Medical University, Xuzhou 221004, China; mayuin1107@163.com; 2 School of Medicine, Southeast University, Nanjing 210009, China; seulsk1031@163.com (S.L.); luoriwangchao@163.com (Y.L.); humingyuewangwang@163.com (M.H.); hpl@seu.edu.cn (P.H.); 3 Department of Surgery, Klinikumrechts der Isar, School of Medicine, Technical University of Munich (TUM), 81675 Munich, Germany; kongbo81@hotmail.com

**Keywords:** loss of heterozygosity, *Kras^G12D^*, HIF-2α, c-Myc, glutamine metabolism

## Abstract

Loss of heterozygosity (LOH) for *KRAS*, in which a wild-type *KRAS* allele is progressively lost, promotes invasive and migratory abilities of pancreatic ductal adenocarcinoma (PDAC) cells and tissues. Moreover, the occurrence of *Kras^G12D^*-LOH activates nonclassical glutamine metabolism, which is related to the malignant behavior of PDAC cells. Herein, we aim to demonstrate the regulatory link between hypoxia-inducible factor-2α (HIF-2α) and glutamine metabolism that mediates malignant phenotypes in *Kras^G12D^*-LOH PDAC cells. HIF-2α-shRNA knockdown lentivirus transfection and metabolite analysis were performed in *Kras^G12D^*-LOH and *Kras^G12D^* cell lines, respectively. Cell proliferation, migration, and invasion were examined using Cell Counting Kit-8, colony formation, and Transwell assays. Cell cycle phase and apoptosis were determined using flow cytometry. Western blotting and real-time quantitative PCR were also performed. Additionally, a subcutaneous xenograft mouse model was established. LOH stimulated HIF-2α activity and transactivated c-Myc, which has a central regulatory effect on glutamine metabolism independent of hypoxia. Meanwhile, HIF-2α silencing repressed *Kras^G12D^*-LOH PDAC cell proliferation, invasion, and migration. HIF-2α knockdown inhibited glutamine uptake and GOT1 expression via a c-Myc-dependent pathway. Collectively, *Kras^G12D^*-LOH can activate HIF-2α to regulate c-Myc-mediated glutamine metabolism and promote malignant phenotypes. Moreover, targeting HIF-2α-c-Myc regulated nonclassical glutamine metabolism, providing a new therapeutic perspective for *Kras^G12D^*-LOH PDAC.

## 1. Introduction

Pancreatic cancer is one of the most common malignant gastrointestinal cancers and is characterized by late diagnosis, early metastasis, lack of a specific targeted treatment, and poor prognosis [1]. Moreover, the prognosis of pancreatic ductal adenocarcinoma (PDAC) has not significantly improved over the past few decades; it has a five-year survival rate of only 6%. Moreover, the specific pathogenesis of pancreatic cancer remains unclear [2,3]. Patients with PDAC usually have no obvious symptoms and are diagnosed at an advanced stage of the disease, which is the primary cause of the associated low survival rate [4,5]. Diagnosed patients are eligible for surgery, chemotherapy, radiotherapy, and immunotherapy; however, there is currently a lack of effective targeted therapies [6]. As such, research has focused on the molecular mechanisms of pancreatic cancer, which may be of great significance for accurate diagnosis, efficient treatment, and improved prognosis.

**Figure 1:**
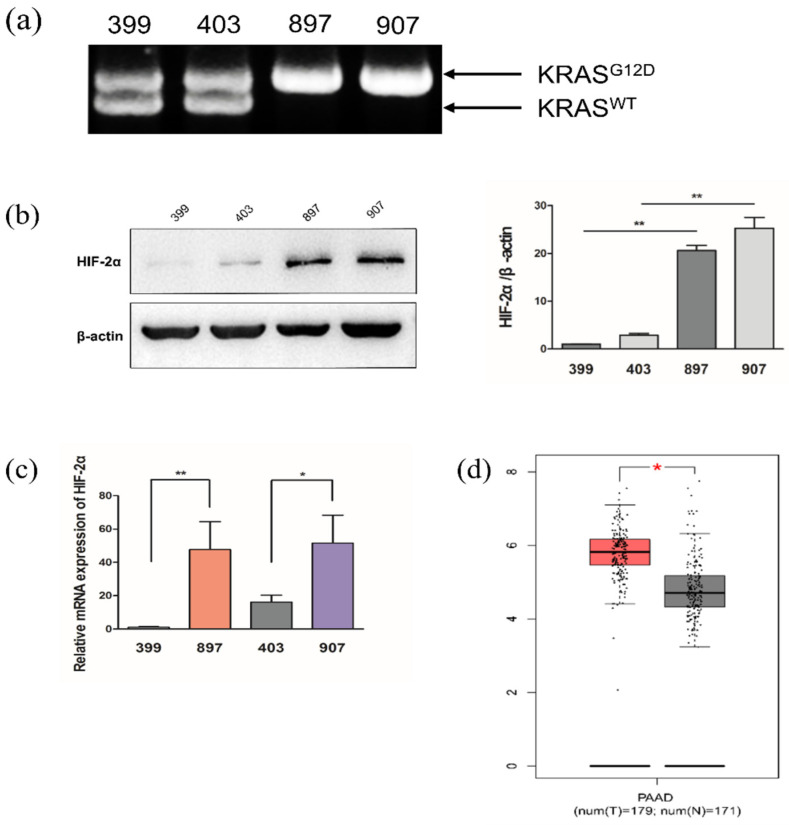
HIF-2α expression in each experimental cell line and pancreatic cancer tissue. (**a**) PCR analysis showed that *Kras^G12D^*-LOH is expressed in 897 and 907 cell lines, but not in the 399 and 403 cell lines. (**b**) Relative expression of HIF-2α in *Kras^G12D^*-loss of heterozygosity (LOH) cells (897 and 907 cells) and *Kras^G12D^* cells (399 and 403 cells) under normoxia. (**c**) Relative mRNA expression of HIF-2α in *Kras^G12D^*-LOH cells (897 and 907 cells) and *Kras^G12D^* cells (399 and 403 cells) under normoxia. (**d**) Relative mRNA levels of HIF-2α expressed in pancreatic tumor tissues and normal pancreatic tissues from the Gene Expression Profiling Interactive Analysis database. Data are expressed as the mean ± standard deviation (SD), based on three independent experiments. * *p* < 0.05; ** *p* < 0.01; PAAD pancreatic adenocarcinoma.

Current studies indicate that pancreatic cancer is mainly regulated by tumor-suppressor gene inactivation and oncogene activation [7]. The *KRAS* gene is one of the most frequently mutated oncogenes in many cancers, and can be found in nearly all PDAC cases [8,9,10]. *KRAS* belongs to a class of genes that encodes guanosine triphosphatase and regulates downstream signaling pathways through growth factor receptors [11]. In addition to *KRAS*, *TP53*, *CDKN2A*, and *SMAD4* tumor suppressor genes are significantly mutated in pancreatic cancer [12,13,14]. Moreover, loss of heterozygosity (LOH), which refers to the transformation of a heterozygous allele to a homozygous state, is a common oncogenic mutation associated with the occurrence and development of various tumors [15,16]. In our previous experiment, we found that *Kras^G12D^*-LOH exists in transgenic mice and human pancreatic cancer cells, wherein heterozygous PDAC cells lose their wild-type *KRAS* allele through mutation (Figure 1a). Furthermore, *Kras^G12D^*-LOH PDAC cells have a stronger ability to proliferate and invade than PDAC cells without LOH [17,18,19]. The specific downstream-signaling mechanism of *Kras^G12D^*-LOH responsible for its high proliferation and invasion remains unclear and requires further study.

Warburg defined tumors as metabolic diseases in the 1920s. It was suggested that even under aerobic conditions, glucose does not participate in the tricarboxylic acid cycle but instead obtains adenosine triphosphate through lactic acid formation via aerobic glycolysis [20]. Glutamine (Gln) is a nonessential amino acid abundant in plasma and contributes to cancer cell genesis, proliferation, and metastasis [21,22]. Gln metabolism can provide carbon and nitrogen sources for tumor cells and participates in the synthesis of biological macromolecules, including lipids, proteins, and other amino acids, which play an important role in the maintenance of cell redox homeostasis, activation of cell-signaling pathways, and regulation of glucose and lipid metabolism [23]. Previous studies have shown that PDAC cells are heavily dependent on Gln metabolism for their growth needs and highly sensitive to Gln deprivation [24,25]. Moreover, unlike other tumor cells entering the tricarboxylic acid cycle by glutamate dehydrogenase-mediated deamination, glutamate is converted to aspartate by aspartate aminotransferase (GOT1) in PDAC cells. Glutamate-derived aspartate is transferred to the cytoplasm to generate oxaloacetate, which in turn produces malate by malate dehydrogenase (MDH1) and pyruvate by malic enzyme (ME1). The reduced nicotinamide adenine dinucleotide phosphate (NADPH) that is generated to restore oxidized glutathione during this pathway is vital for maintaining the intracellular redox balance. Pancreatic tumor cells metabolize Gln via a nonclassical pathway, which is mediated by *KRAS* [26]. In our previous study, LOH for *Kras^G12D^* stimulated glutaminolysis in PDAC cells [19]. *Kras^G12D^*-LOH can increase protein and mRNA levels of related molecular markers, including GLS1, GOT1, GOT2, MDH1, and ME1, under both normoxic and hypoxic conditions [19]. However, the specific mechanism underlying *Kras^G12D^*-LOH and Gln metabolism remains unclear.

Hypoxia-inducible factors (HIFs) serve as the main regulators of a series of molecular biological changes in tumor cells, including angiogenesis, erythropoiesis, glycolysis, and subsequent tumor progression [27]. HIF-α can be regulated by oxygen and consists of three subunits: HIF-1α, HIF-2α, and HIF-3α [28]. Current studies have shown that HIF-2α correlates with normal cell proliferation, differentiation, energy metabolism, erythropoiesis, stem cell maintenance, inflammation, tumor proliferation, metastasis, and angiogenesis [29]. HIF-2α is hydroxylated by prolyl hydroxylase and degrades rapidly under aerobic conditions after binding to von Hippel–Lindau tumor suppressor protein (pVHL) [30]. The degradation of HIF-α is inhibited in response to hypoxia, and HIF-2α can initiate transcription of the target gene by binding to the hypoxia response elements of target genes to form a transcription-initiation complex [31,32]. Meanwhile, intracellular lactate signaling, which imitates a response to hypoxia, reportedly promotes Gln metabolism through the HIF-2α-signaling pathway in oxidative cancer cells in a manner dependent on c-Myc activation [33]. Similarly, studies have shown that HIF-2α affects PDAC cell growth by regulating Gln metabolism under prolonged hypoxia [34].

Our aim was to determine the role of HIF-2α in the *Kras^G12D^*-LOH-mediated malignant phenotype and examine the relationship between *Kras^G12D^*-LOH and HIF-2α-regulated Gln metabolism that occurs independently of hypoxia.

## 2. Results

### 2.1. Kras^G12D^-LOH Elevated HIF-2α Expression in PDAC Cells at Both Protein and mRNA Levels

To confirm the regulatory role of HIF-2α in Gln metabolism, we investigated HIF-2α protein and mRNA expression under normoxic conditions. Notably, both HIF-2α protein and mRNA expression were significantly elevated in *Kras^G12D^*-LOH PDAC cells compared with *Kras^G12D^* PDAC cells without LOH under normoxic conditions (*p* < 0.05, *p* < 0.01; Figure 1b,c). We used the Gene Expression Profiling Interactive analysis (GEPIA) database (http://gepia.cancer-pku.cn/ accessed on 16 April 2021) to analyze the HIF-2α mRNA level in pancreatic cancer tissues and normal pancreatic tissues and found that HIF-2α mRNA levels were significantly higher in pancreatic cancer tissues than in normal pancreatic tissues (*p* < 0.05; Figure 1d).

### 2.2. Kras^G12D^-LOH Triggered HIF-2α-Dependent c-Myc Activation

**Figure 2:**
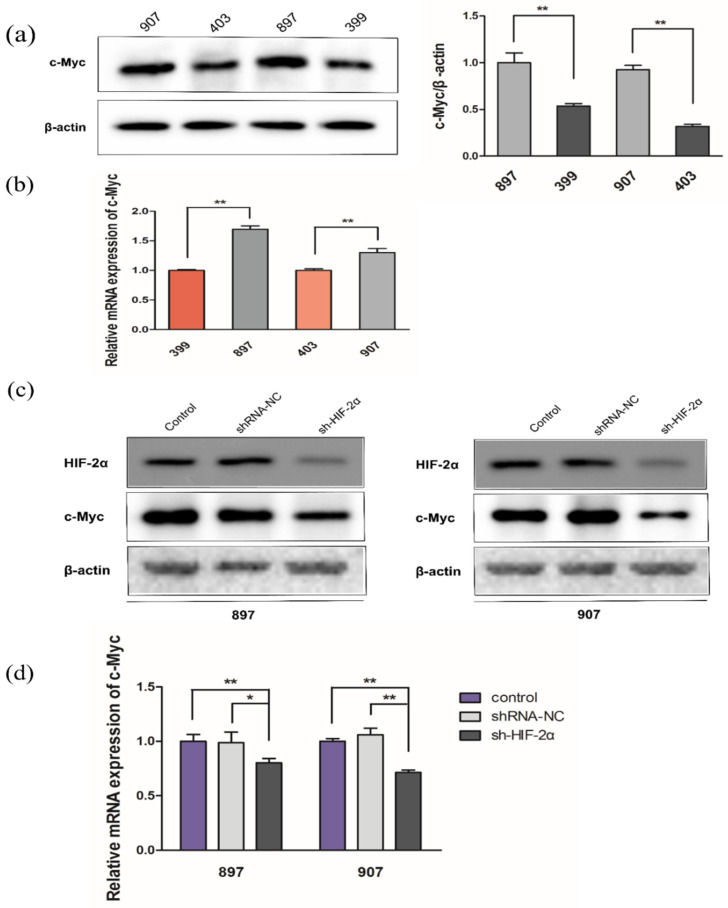
*Kras^G12D^*-LOH induces HIF-2α-dependent c-Myc activation. (**a**) Relative protein expression of c-Myc in *Kras^G12D^*-LOH pancreatic ductal adenocarcinoma (PDAC) cells (897 and 907 cells) and *Kras^G12D^* PDAC cells (399 and 403 cells). (**b**) Relative mRNA expression of c-Myc in *Kras^G12D^*-LOH PDAC cells (897 and 907 cells) and *Kras^G12D^* PDAC cells (399 and 403 cells). (**c**) Relative protein expression of c-Myc in sh-HIF-2α-transfected *Kras^G12D^*-LOH PDAC cells (897 and 907 cells) compared with that in shRNA-NC and control groups. (**d**) Relative mRNA expression of c-Myc in sh-HIF-2α-transfected *Kras^G12D^*-LOH PDAC cells (897 and 907 cells) compared with that in the shRNA-NC and control groups. Data are expressed as the mean ± SD, based on three independent experiments. * *p* < 0.05; ** *p* < 0.01.

Mitochondrial glutaminase and Gln metabolism can be enhanced by c-Myc via suppression of miR-23 [35]. However, a specific link between LOH and c-Myc has not yet been reported. Notably, c-Myc protein and mRNA levels were significantly higher in *Kras^G12D^*-LOH cells than in *Kras^G12D^* cells without LOH (*p* < 0.01; Figure 2a,b). *Kras^G12D^*-LOH promoted c-Myc activity, and HIF-2α knockdown repressed c-Myc expression both at the protein and mRNA levels (*p* < 0.01; Figure 2c,d). The LOH-induced elevation of c-Myc expression was blocked by HIF-2α silencing. According to these findings, LOH activated HIF-2α, which further stabilized c-Myc activity via a signaling pathway independent of hypoxia.

### 2.3. LOH-Activated HIF-2α-c-Myc Signaling Enhanced Noncanonical Gln Metabolism

**Figure 3:**
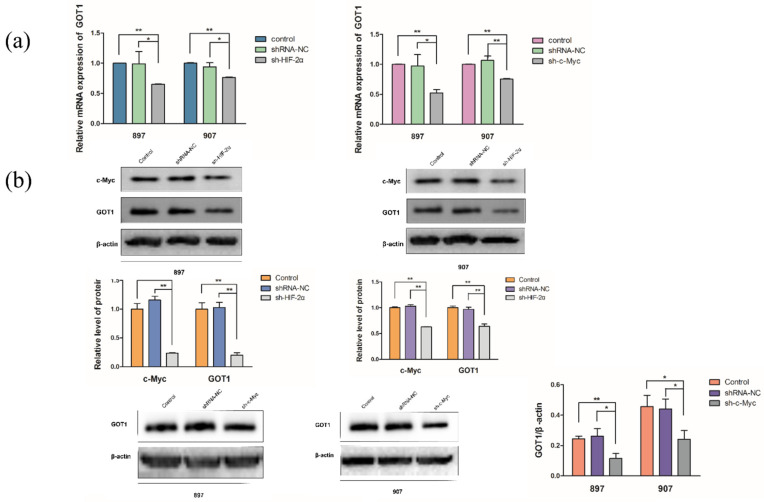

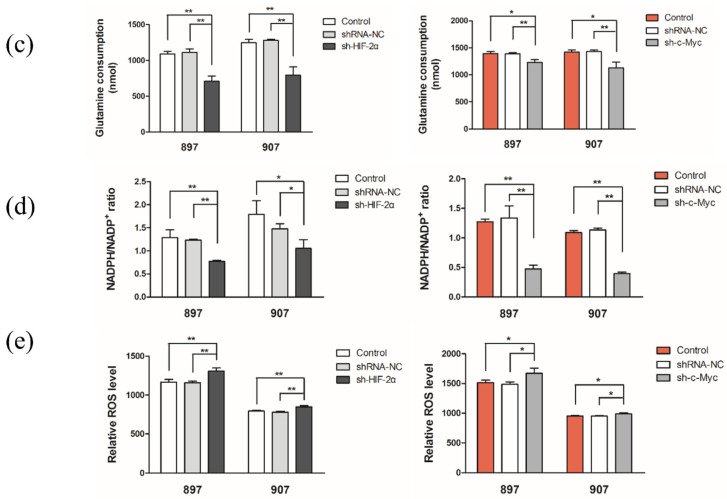
*Kras^G12D^*-LOH-activated HIF-2α-c-Myc signaling regulates glutamine metabolism. (**a**) Relative mRNA expression of GOT1 in sh-HIF-2α- or sh-c-Myc-transfected *Kras^G12D^*-LOH cells (897 and 907 cells) compared with that in the shRNA-NC and control groups. (**b**) Relative protein expression of GOT1 in sh-HIF-2α- or sh-c-Myc-transfected *Kras^G12D^*-LOH cells (897 and 907 cells) compared with that in the shRNA-NC and control groups. (**c**) Glutamine consumption rate. (**d**) NADPH:NADP^+^ ratio. (**e**) Reactive oxygen species (ROS) levels. Data are expressed as the mean ± SD, based on three independent experiments. * *p* < 0.05; ** *p* < 0.01.

To determine the role of HIF-2α and c-Myc in Gln metabolism, we transfected shRNA lentivirus targeted with c-Myc and shRNA targeted with HIF-2α into *Kras^G12D^* and *Kras^G12D^*-LOH cells, respectively. HIF-2α or c-Myc silencing repressed GOT1 protein and mRNA levels in *Kras^G12D^*-LOH cells (*p* < 0.05 and *p* < 0.05, respectively; Figure 3a,b). LOH-activated GOT1 expression in *Kras^G12D^*-LOH PDAC cells was independently repressed by sh-HIF-2α or sh-c-Myc compared with that in the shRNA-NC and control groups. HIF-2α or c-Myc knockdown significantly decreased Gln consumption in *Kras^G12D^*-LOH PDAC cells (*p* < 0.01 and *p* < 0.05; Figure 3c). Moreover, we observed a reduction in NADPH levels in sh-HIF-2α- and sh-c-Myc *Kras^G12D^*-LOH PDAC cells; specifically, improved NADP^+^ ratios and reactive oxygen species (ROS) levels were observed (*p* < 0.05, and *p* < 0.05, respectively; Figure 3d,e). These findings suggest that inhibition of HIF-2α attenuated nonclassical Gln metabolism in *Kras^G12D^*-LOH pancreatic cancer cells, possibly in a c-Myc-dependent manner.

### 2.4. HIF-2α Knockdown Impaired Kras^G12D^ and Kras^G12D^-LOH PDAC Cell Growth

**Figure 4:**
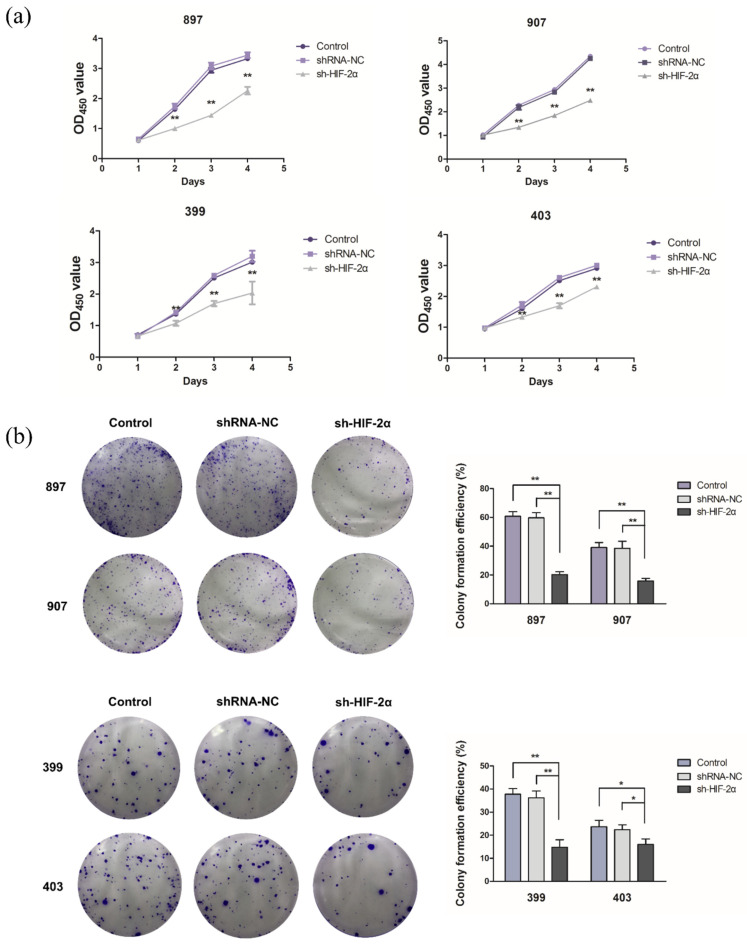
HIF-2α knockdown inhibits the proliferation of *Kras^G12D^* and *Kras^G12D^*-LOH PDAC cells. *Kras^G12D^* and *Kras^G12D^*-LOH PDAC cells were transfected with sh-HIF-2α and shRNA-NC. (**a**) Cell viability according to CCK-8 assay. (**b**) Cell proliferation measured by colony formation assay. Data are expressed as the mean ± SD, based on three independent experiments. * *p* < 0.05; ** *p* < 0.01.

As we confirmed that HIF-2α had a regulatory effect on nonclassical Gln metabolism, we further investigated whether the malignant behavior of *Kras^G12D^*-LOH PDAC cells was associated with HIF-2α knockdown. CCK-8 analysis revealed that HIF-2α silencing reduced the proliferation rate of sh-HIF-2α *Kras^G12D^* cells and *Kras^G12D^*-LOH cells (*p* < 0.01; Figure 4a). Similarly, a colony formation assay showed a decreased colony number in *Kras^G12D^* PDAC cells and *Kras^G12D^*-LOH PDAC cells (*p* < 0.05 and *p* < 0.01, respectively; Figure 4b). Overall, HIF-2α knockdown halted the proliferation of both *Kras^G12D^*-LOH and *Kras^G12D^* PDAC cells.

### 2.5. HIF-2α Knockdown Suppressed Invasion and Migration of Kras^G12D^ and Kras^G12D^-LOH PDAC Cells

**Figure 5:**
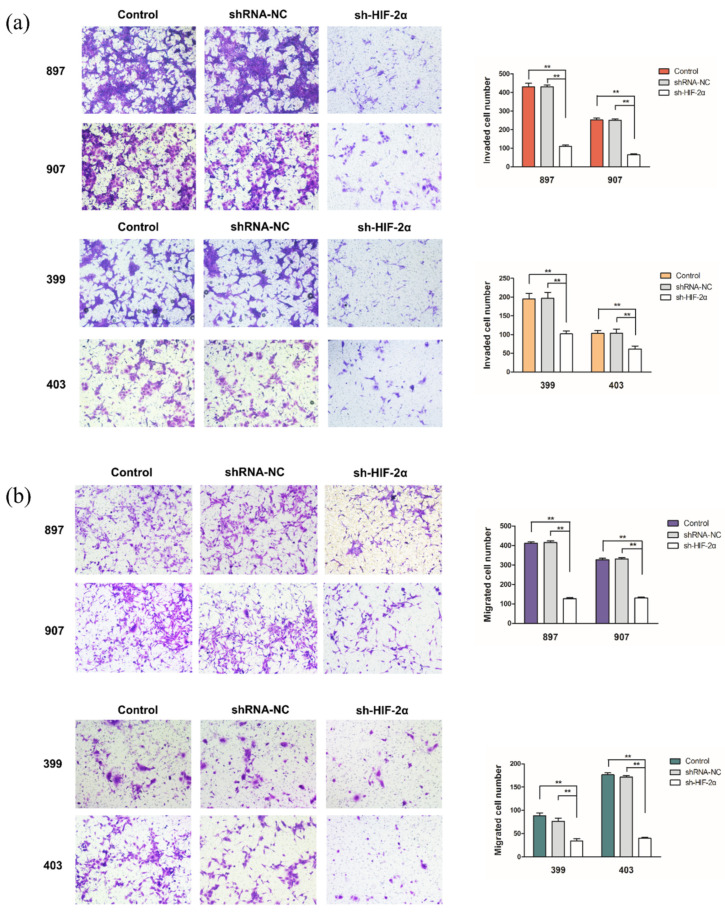
HIF-2α knockdown inhibits the invasion and migration of *Kras^G12D^* and *Kras^G12D^*-LOH PDAC cells. *Kras^G12D^* and *Kras^G12D^*-LOH PDAC cells were transfected with sh-HIF-2α and shRNA-NC. (**a**,**b**) The invasion and migration capacity of *Kras^G12D^* and *Kras^G12D^*-LOH cells according to Transwell assays. Data are expressed as the mean ± SD, based on three independent experiments. ** *p* < 0.01.

According to the results of Transwell assay, HIF-2α silencing reduced the invasion of *Kras^G12D^* and *Kras^G12D^*-LOH cells (*p* < 0.01; Figure 5a). Consistently, the sh-HIF-2α group had a lower number of invaded *Kras^G12D^* and *Kras^G12D^*-LOH cells than the shRNA-NC and control groups (*p* < 0.01; Figure 5b). These findings demonstrate that HIF-2α silencing can inhibit the migration and invasion of *Kras^G12D^* and *Kras^G12D^*-LOH PDAC cells.

### 2.6. HIF-2α Knockdown Increased Cell Cycle Arrest and Apoptosis of Kras^G12D^ and Kras^G12D^-LOH PDAC Cells

**Figure 6:**
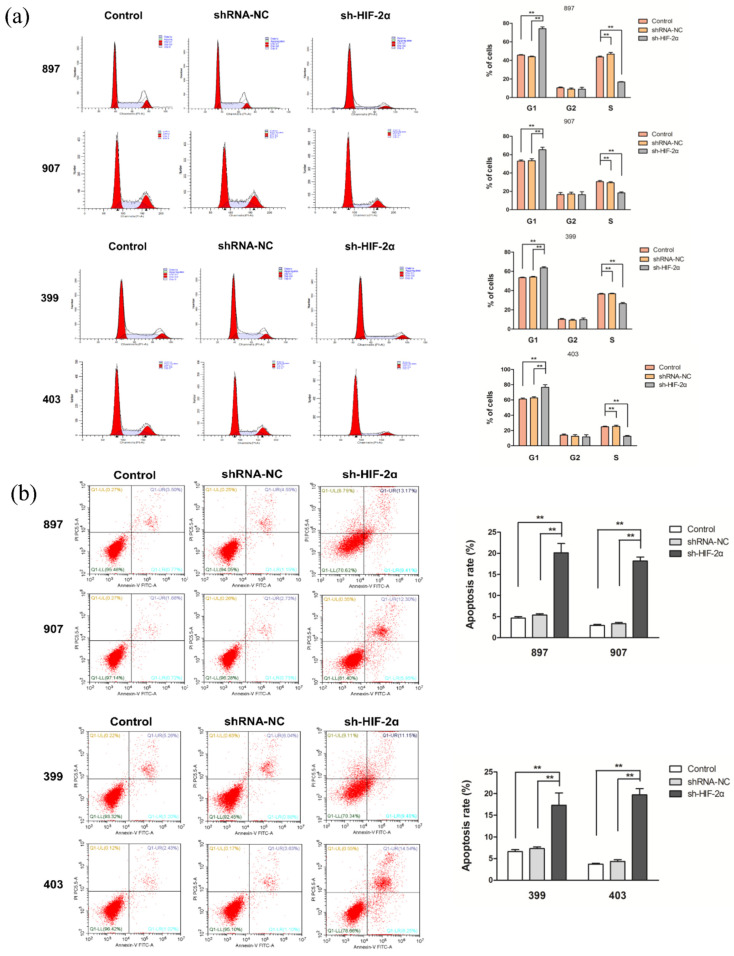
HIF-2α knockdown inhibits cell cycle progression and stimulates *Kras^G12D^* and *Kras^G12D^*-LOH PDAC cell apoptosis. *Kras^G12D^* and *Kras^G12D^*-LOH PDAC cells were transfected with sh-HIF-2α and shRNA-NC. (**a**) Cell cycle distribution of *Kras^G12D^* and *Kras^G12D^*-LOH cells. (**b**) Apoptosis rate of *Kras^G12D^* and *Kras^G12D^*-LOH cells. Data are expressed as the mean ± SD, based on three independent experiments. ** *p* < 0.01.

The cell cycle assay results showed that the proportion of cells in the S phase was significantly lower in sh-HIF-2α *Kras^G12D^*-LOH and sh-HIF-2α *Kras^G12D^* groups than in the control and shRNA-NC groups, whereas HIF-2α knockdown induced G1 phase arrest (*p* < 0.01; Figure 6a). In addition, the sh-HIF-2α groups of *Kras^G12D^*-LOH and *Kras^G12D^* cells had an increased apoptosis rate (*p* < 0.01; Figure 6b). These results confirmed that the proliferative capacity of *Kras^G12D^* and *Kras^G12D^*-LOH PDAC cells was inhibited following HIF-2α knockdown.

### 2.7. Role of HIF-2α in Kras^G12D^ and Kras^G12D^-LOH PDAC Cells In Vivo

**Figure 7:**
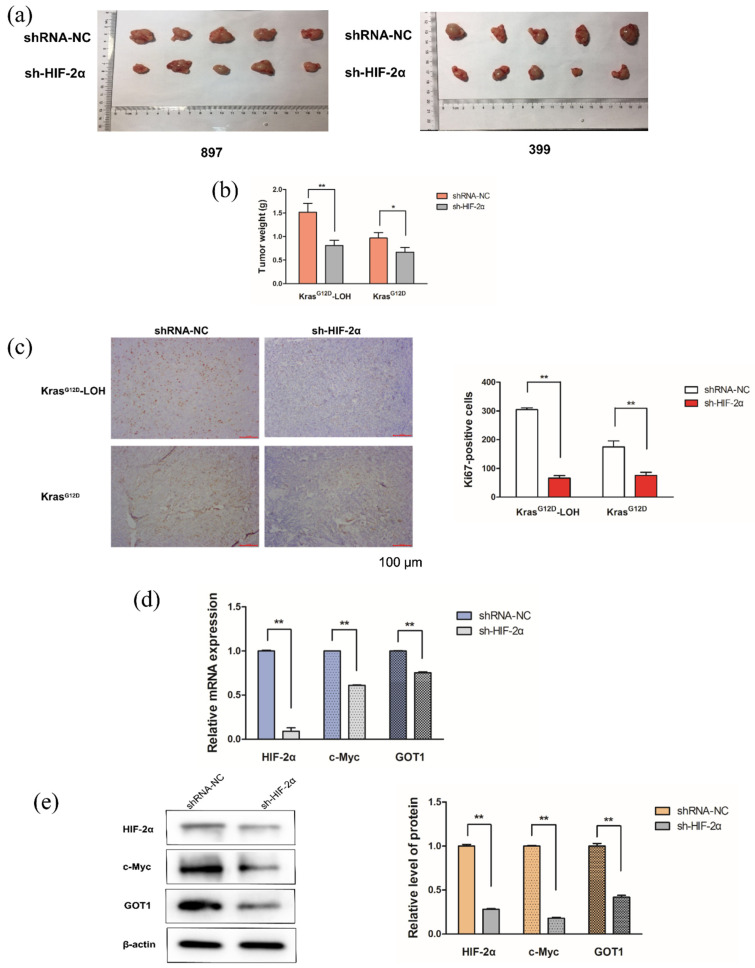
HIF-2α knockdown represses the malignant phenotype of PDAC cells in vivo and regulates the nonclassical glutamine metabolism. (**a**) Comparison between tumors removed from *Kras^G12D^*-LOH and *Kras^G12D^* mice. (**b**) Weight of tumors removed from the *Kras^G12D^*-LOH and *Kras^G12D^* mice. (**c**) Ki67 immunostaining of tumors. (**d**) Relative mRNA expression of c-Myc and GOT1 in *Kras^G12D^*-LOH tumor tissues. (**e**) Relative protein expression of c-Myc and GOT1 in the *Kras^G12D^*-LOH tumor tissues. Data are expressed as the mean ± SD, based on three independent experiments. * *p* < 0.05; ** *p* < 0.01.

We further demonstrated the exact role of HIF-2α in malignant behavior and nonclassical Gln metabolism in vivo. After subcutaneous injection of HIF-2α-targeted shRNA-infected cells into nude mice, we observed that the weight of the removed tumor in the sh-HIF-2α group was significantly lower than that in the shRNA-NC group (*p* < 0.05; Figure 7a,b). In addition, immunohistochemistry revealed that HIF-2α silencing decreased the number of Ki67-positive PDAC cells compared with that in the shRNA-NC group (*p* < 0.01; Figure 7c). Consistent with the in vitro results, we noted that HIF-2α silencing inhibited LOH-induced c-Myc and GOT1 expression at both mRNA and protein levels (*p* < 0.01; Figure 7d,e). Overall, these results demonstrate that HIF-2α silencing may repress the malignant phenotype of *Kras^G12D^*-LOH PDAC cells in vivo, and HIF-2α-c-Myc signaling may regulate nonclassical Gln metabolism.

## 3. Discussion

This study mainly explored the role of HIF-2α in the *Kras^G12D^*-LOH cell-mediated malignant phenotype of pancreatic cancer. In particular, we investigated the downstream targets of *Kras^G12D^*-LOH and found that the protein and mRNA levels of HIF-2α in *Kras^G12D^*-LOH PDAC cells were significantly higher than those in *Kras^G12D^* PDAC cells. These results suggest that HIF-2α plays a key role in regulating the malignant phenotype and Gln metabolism in *Kras^G12D^*-LOH pancreatic cancer.

Hypoxia is among the fundamental factors regulating the HIF-signaling pathway [36]. Some studies have found that malignant tumor cells can promote the activation of the HIF-signaling pathway by inducing HIF-2α mRNA transcription, maintaining protein stability and regulating upstream and downstream target genes in various pathways [37,38]. In addition, mutation of VHL or inactivation of prolyl hydroxylase can promote HIF-2α expression [39]. Prolyl hydroxylation can be inhibited by ROS, nitric oxide, and specific oncogenes, such as activated RAS and v-Src [40,41]. The PI3K/AKT/mammalian target of rapamycin (mTOR) pathway activates HIF signaling by increasing HIF-α subunit protein translation [42]. Similarly, in the present study, we found that HIF-2α expression was significantly higher in *Kras^G12D^*-LOH pancreatic cancer cells than in *Kras^G12D^* pancreatic cancer cells under normoxia. Furthermore, the occurrence of LOH in *Kras^G12D^* PDAC cells was found to promote HIF-2α expression. The LOH for *Kras^G12D^* stabilizes HIF-2α expression, which was confirmed in the experimental 897 and 907 cell lines without hypoxia signaling. We successfully constructed sh-HIF-2α and shRNA-NC lentiviruses to transfect *Kras^G12D^*-LOH and *Kras^G12D^* pancreatic cancer cells. The results showed that inhibition of HIF-2α can suppress the proliferation, migration, and invasion of *Kras^G12D^*-LOH and *Kras^G12D^* pancreatic cancer cells. Moreover, HIF-2α silencing can block the cell cycle and promote apoptosis. Therefore, HIF-2α plays a crucial role in the regulation of malignant phenotypes in *Kras^G12D^*-LOH and *Kras^G12D^* pancreatic cancer cells.

Pancreatic cancer cells metabolize Gln via a nonclassical pathway. Gln-derived glutamate yields aspartate through GOT1, which is then transferred to the cytoplasm to produce oxaloacetate, malic acid, and pyruvate by MDH1 and ME1 [26]. In contrast to those in *Kras^G12D^* pancreatic cancer cells, the Gln uptake rate and NADPH/NADP^+^ ratio of *Kras^G12D^*-LOH pancreatic cancer cells were significantly decreased after HIF-2α downregulation, whereas ROS levels increased compared with those in the control and shRNA-NC groups. HIF-2α-mediated Gln metabolism was more apparent in *Kras^G12D^*-LOH PDAC cells. In addition, LOH activated c-Myc protein and mRNA levels, which had a regulatory effect on Gln metabolism; moreover, HIF-2α downregulation inhibited c-Myc expression. The suppression of HIF-2α or c-Myc significantly decreased GOT1 levels in the Gln metabolic pathway compared with those in the control group. The occurrence of LOH first increases HIF-2α expression; HIF-2α then enhances c-Myc activity, which regulates Gln metabolism in a pathway independent of hypoxia. These results suggest that HIF-2α knockdown can significantly repress Gln metabolism in *Kras^G12D^*-LOH pancreatic cancer cells in a manner dependent on c-Myc, without a significant change in Gln metabolism in *Kras^G12D^* pancreatic cancer cells. *Kras^G12D^*-LOH can promote Gln metabolism in *Kras^G12D^*-LOH pancreatic cancer cells by regulating the HIF-2α-c-Myc pathway, and HIF-2α plays an important role in regulating Gln metabolism in *Kras^G12D^*-LOH pancreatic cancer cells. Furthermore, the regulatory trend of nonclassical Gln metabolism is consistent with the malignant behavior of *Kras^G12D^*-LOH pancreatic cancer cells, suggesting that *Kras^G12D^*-LOH may regulate the malignant phenotype of pancreatic cancer through HIF-2α/c-Myc-mediated Gln metabolism. However, the precise mechanism by which the occurrence of LOH stimulates the HIF-2α pathway and hypoxia signaling requires further exploration in future research. In addition, clinical data must be collected and analyzed to confirm the regulatory role of HIF-2α in *Kras^G12D^*-LOH PDAC. Although HIF-2α knockdown results in a similar phenotype in *Kras^G12D^* and *Kras^G12D^*-LOH cells, this is not the case for *Kras^G12D^*-LOH cells. Because *Kras^G12D^*-LOH cell lines have a higher proliferation and metastasis capability, they were more effective at inhibiting the malignant phenotype than *Kras^G12D^* cells after HIF-2α knockdown. In addition, the regulation mechanism of HIF-2α in *Kras^G12D^* cell phenotypes requires further investigation. We hypothesize that additional pathways regulate the malignant phenotype of *Kras^G12D^* and *Kras^G12D^*-LOH cell lines simultaneously.

In conclusion, the findings of this study suggest that HIF-2α silencing in *Kras^G12D^*-LOH pancreatic cancer cells significantly decreases Gln metabolism and inhibits cell proliferation and invasion. Meanwhile, although downregulation of HIF-2α expression has minimal effects on Gln metabolism in *Kras^G12D^* pancreatic cancer cells, it inhibits cell proliferation and invasion, suggesting that *Kras^G12D^*-LOH regulates the malignant biological behavior of pancreatic cancer in HIF-2α-mediated Gln metabolism. Therefore, HIF-2α can be activated by *Kras^G12D^*-LOH to regulate Gln metabolism and participate in malignant phenotypes.

## 4. Materials and Methods

### 4.1. Cell Culture and Transfection

*Kras^G12D^*-(herein designated as 399 and 403 cells) and *Kras^G12D^*-LOH cells (herein designated as 897 and 907 cells) were obtained from transgenic mice, generously provided by Bo Kong (Technical University of Munich, Munich, Germany). As previously described, all four cell lines are were isolated from transgenic p48^Cre/+^; LSL-*Kras^G12D/+^*; Tsc1^fl/+^ mice [43]. The four experimental cell lines were cultured as described previously [19]. Four experimental PDAC cells were seeded in 96-well plates (1 × 10^4^ cells/well) and cultured until reaching 40–60% confluence. Transfection experiments were conducted using lentiviruses (GenePharma Co., Shanghai, China) targeting HIF-2α or c-Myc with the addition of 5 μg/mL polybrene, following the manufacturer’s instructions. Fresh medium containing puromycin (2 μg/mL) was added daily for 2–3 weeks. HIF-2α shRNA sequences were as follows: (sense, 5′–3′) CGACAGAATCTTGGAACTGAT and (antisense, 5′–3′) ATCAGTTCCAAGATTCTGTCG. c-Myc shRNA sequences were as follows: (sense, 5′–3′) GCCTACATCCTGTCCATTCAA and (antisense, 5′–3′) TTGAATGGACAGGATGTAGGC.

### 4.2. Polymerase Chain Reaction (PCR)

RNAiso Plus reagent (Takara Biotechnology, Dalian, China) was applied for the isolation of total RNA, and reverse-transcription of the isolated RNA to cDNA was performed using an RT Master Mix for qPCR (MedChemExpress, Monmouth Junction, NJ, USA), strictly following the manufacturer’s instructions. Genomic DNA was extracted using a PureLink^TM^ Genomic DNA Mini Kit (Invitrogen, Carlsbad, CA, USA). Real-time quantitative PCR was performed, and results were analyzed using an ABI 7500 PCR system (Thermo Fisher Scientific, Waltham, MA, USA) using SYBR Green qPCR Master Mix (MedChemExpress). The primers used were as follows: HIF-2α, 5′-ATCCCTATGGACGGCGAG-3′ (forward), and 5′-CAACTGCTGCGGGTACTTAT-3′ (reverse); c-Myc, 5′-AAACGACAAGAGGCGGACAC-3′ (forward) and 5′-TGGTCACGCAGGGCAAAA-3′ (reverse); GOT1, 5′-CGAGTACCTGCCCATCCTG-3′ (forward) and 5′-ACCATCGCCCTAAGAAGTCA-3′ (reverse); and β-actin, 5′-CACCCCATTTGATGTTAGTG-3′ (forward) and 5′-CCATTTGCAGTGGCAAAG-3′ (reverse). In addition, PCR was performed on a StepOnePlus^TM^ Real-Time PCR system to distinguish the wide-type *Kras* and activated *Kras^G12D^* mutant alleles. Genotyping was confirmed by agarose gel electrophoresis. The primers used were as follows: Krasboth, 5′-AGGCCTGCTGAAAATGACTG-3′ (forward), and 5′-TGGT TCCCTAACACCCAGTT-3′(reverse).

### 4.3. Western Blotting

RIPA buffer, which contained a protease inhibitor (Cell Signaling Technology, Danvers, MA, USA), was applied for cell lysis. The protein concentrations were determined using a BCA protein assay kit (KeyGEN BioTECH, Nanjing, China). SDS-PAGE (8–10%) was used to separate the proteins, which were then transferred to PVDF membranes (Merck Millipore, Billerica, MA, USA). The primary antibodies included HIF-2α (1:500, ab109616, Abcam, Cambridge, UK), c-Myc (1:500, #13987, Cell Signaling Technology), GOT1 (1:1000, #14886-1-AP, Proteintech, Rosemont, IL, USA), and β-actin (1:5000, #AP0060, Bioworld, Bloomington, MN, USA). Enhanced chemiluminescence (Merck Millipore) was used to evaluate the signal, and analysis was performed using the ImagePro Plus software (Media Cybernetics, Rockville, MD, USA). Each target gene protein was semi-quantitatively estimated, compared with β-actin as an internal loading control.

### 4.4. Measurement of Gln Consumption, the NADPH:NADP^+^ Ratio, and Intracellular ROS Levels

Four experimental cell lines were seeded in 6-well (10^5^/well) plates for 48 h. A colorimetric assay kit (Biovision, Milpitas, CA, USA), NADPH/NADP^+^ assay kit (Jiancheng Bioengineering Institute, Nanjing, China), and ROS detection kit (Jiancheng Bioengineering Institute) were used to measure Gln consumption, the NADPH:NADP^+^ ratio, and intracellular ROS levels, respectively, according to the manufacturers’ instructions. The specific methods were described previously [19].

### 4.5. Cell Viability, Colony Formation, Transwell, Cell Cycle, and Apoptosis Assays

The relevant methods are described in our previous studies [19] and the Supplementary Materials.

### 4.6. Xenograft Mouse Model

Female BALB/c nude mice (4 ± 1 week old, 14 ± 5 g) were injected with 200 mL (1 × 10^6^ cells) of sh-HIF-2α-transfected *Kras^G12D^* and *Kras^G12D^*-LOH cells. Additionally, shRNA-NC cells (5 mice per group) were subcutaneously injected into the right axillary fossa. Subsequently, each animal was sacrificed by cervical dislocation 14 days after inoculation, and the tumors were retrieved. The animal experiments complied with the ARRIVE guidelines and were approved by the Ethics Committee on Animal Care and Use of Southeast University (No. 20200101003).

### 4.7. Immunohistochemistry

The tumors retrieved from BALB/c nude mice were fixed in formalin and embedded in paraffin; 4 μm-thick sections were prepared. The sections were first incubated with a primary antibody for Ki67 (1:200, 12202T, Cell Signaling Technology, Boston, MA, USA) overnight at 4 °C and subsequently incubated at 37 °C for 30 min with a corresponding secondary antibody (1:2000, 111-035-003, Jackson ImmunoResearch Inc, West Grove, PA, USA) at 37 °C. Finally, the sections were re-stained with hematoxylin and observed under a microscope (Nikon Eclipse, Tokyo, Japan).

### 4.8. GEPIA Database Analysis

Analysis of HIF-2α mRNA expression in pancreatic cancer tissues and normal pancreatic tissues was conducted using the GEPIA (http://gepia.cancer-pku.cn/ accessed on 16 April 2021) database.

### 4.9. Statistical Analysis

All experiments were independently conducted at least three times. The results are presented as the mean ± standard deviation (SD). The obtained experimental data were analyzed using one-way analysis of variance and Student’s *t*-test. The SPSS software (IBM Corp., Armonk, NY, USA) was used for all experimental analyses. *p*-values < 0.05 were considered statistically significant.

## Data Availability

The data presented in this study are available on request from the corresponding author.

## Supplementary Materials

The following supporting information can be downloaded at: www.mdpi.com/article/10.3390/ijms23126697/s1.
