# Supplementary material for: Loss of Heterozygosity for KrasG12D Promotes Malignant Phenotype of Pancreatic Ductal Adenocarcinoma by Activating HIF-2α-c-Myc-Regulated Glutamine Metabolism
Source: Int J Mol Sci. 2022 Jun 15;23(12):6697. doi: 10.3390/ijms23126697 (PMC9224498; doi:10.3390/ijms23126697)
Supplement: Supplementary file 1 [file ijms-23-06697-s001.zip › ijms-1738007-supplementary.pdf]

Supplementary Methods

Cell viability assay

Four experimental cell lines were seeded in 96-well plates at 3 × 10^3 cells/well (100 μL/well) and then incubated for 0, 24, 48, 72, or 96 h. Next, upon removing the culture medium, 10 μL of Cell Counting Kit-8 (CCK-8) solution (Dojindo, Tokyo, Japan) was added to each well. After incubating for 4 h at 37 °C, the optical density was determined at 450 nm (OD_{450}) with a spectrophotometric plate reader (Multiskan FC, Thermo Fisher Scientific, Waltham, MA, USA). Next, the relative percentage of viable cells was calculated as follows: (OD_{450} at detection time/OD_{450} at 0 h) × 100.

Colony formation assay

Four experimental cell lines were seeded in 6-well plates at a density of 300 cells/well and incubated at 37 °C for 10 days. Next, 4% paraformaldehyde was used to fix the colonies for 30 min before staining with 0.5% crystal violet for 30 min. A cluster comprising a minimum of 50 cells was considered a colony.

Cell invasion assay

Transwell chambers (8 μm pores; Corning, Corning, NY, USA) were precoated with 50 μL of Matrigel (1:3 mixed with fetal bovine serum [FBS]-free medium; BD Biosciences, Philadelphia, PA, USA). Afterward, 5 × 10^5 cells in FBS-free medium were plated in the upper chambers and 600 μL of medium with 10% FBS was placed in the lower chambers to instigate cell invasion in the upper chambers. Following incubation for 24 h, the cells were removed from the upper chamber using a cotton swab. Paraformaldehyde was used to fix the cells in the bottom chamber for 30 min before staining with 0.5% crystal violet

for 30 min. Next, the number of cells was counted under a microscope (Nikon Eclipse, Tokyo, Japan).

Cell cycle analysis

A cell cycle detection kit (KeyGEN BioTECH, Nanjing, China) was used to assess the cell cycle. Cold phosphate-buffered saline (PBS) was used to harvest and wash the cells. Subsequently, the cell concentrations were adjusted to 1×10^6 cells/mL and fixed in 70% cold ethyl alcohol at 4 °C overnight. The ethyl alcohol was then removed, and RNase A was added. The incubation process was conducted for 30 min at 37 °C, followed by staining with 400 μL of propidium iodide (PI) for 30 min at 4 °C. Flow cytometry (Becton Dickinson, Mountain View, CA, USA) was used to determine cell cycle progression.

Cell apoptosis assay

An Annexin V-FITC apoptosis detection kit (KeyGEN BioTECH) was used to measure cell apoptosis. Frozen PBS was used to wash harvested cells. Next, 400 μL of binding buffer (5 μL of Annexin V-FITC with 5 μL of PI added) was used to resuspend and incubate the cells for 15 min at 25 °C in the dark. Flow cytometry (Becton Dickinson) was performed to assess the data.
